# Supplementary material for: Cooperative dynamics of neighborhood economic status in cities
Source: PLoS One. 2017 Aug 17;12(8):e0183468. doi: 10.1371/journal.pone.0183468 (PMC5560684; doi:10.1371/journal.pone.0183468)

S1 Appendix. Flowchart describing update algorithm. At each iteration of the model, the following sequence of actions is undertaken to generate the dynamics of neighborhood Economic Status.

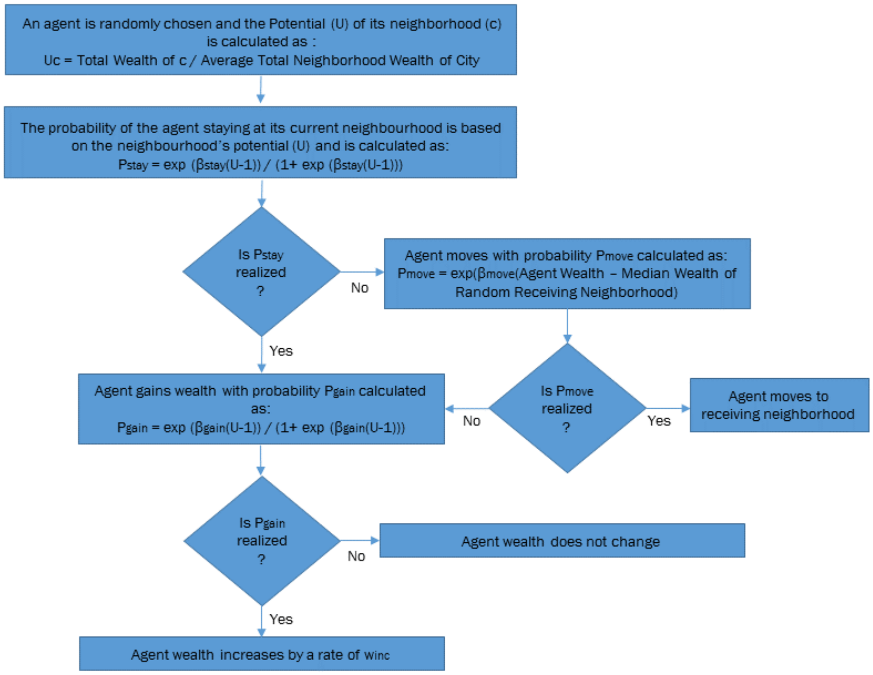

Supplement: S1 Appendix — (PDF) [file pone.0183468.s001.pdf]
