# Supplementary material for: Cooperative dynamics of neighborhood economic status in cities
Source: PLoS One. 2017 Aug 17;12(8):e0183468. doi: 10.1371/journal.pone.0183468 (PMC5560684; doi:10.1371/journal.pone.0183468)
Supplement: S2 Appendix — (PDF) [file pone.0183468.s002.pdf]

S2 Appendix. Additional Charts.

Chart 1: Evolution of RES:

The following figure plots the evolution of RES over time for each of the 8 distinct parameter sets. We find that in each of the 8 plots, RES I and RES IV neighborhoods tend to retain status much more frequently that RES II and RES III neighborhoods. Thus the overall average behavior of RES replicates the behavior of each of the individual parameter set ensemble averages.

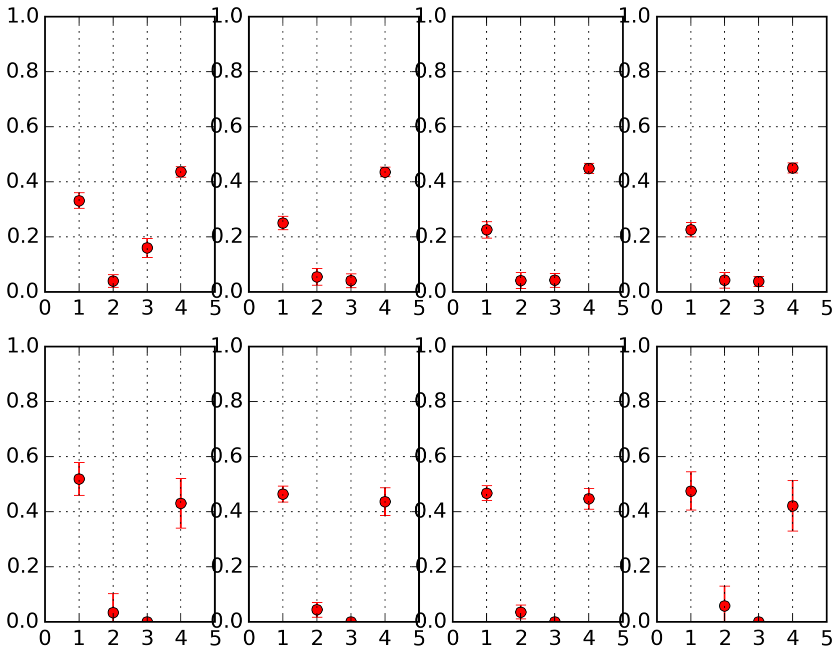

Fig 1. Fraction of neighborhoods of different RES retaining status. Legend: Y-Axis: F = Fraction of neighborhoods with changing RES; X-Axis: X = RES. 1 = RES I, 2 = RES II, 3 = RES III, 4 = RES IV. Top Left: $\beta_{\text{move}} = 10$, wealth configuration = LN ($\mu = 0$, $\sigma = 0.25$); Top 2nd Left: $\beta_{\text{move}} = 100$, wealth configuration = LN ($\mu = 0$, $\sigma = 0.25$); Top 2nd Right: $\beta_{\text{move}} = 1000$, wealth configuration = LN ($\mu = 0$, $\sigma = 0.25$); Top Right: $\beta_{\text{move}} = 100{,}000$, wealth configuration = LN ($\mu = 0$, $\sigma = 0.25$); Bottom Left: $\beta_{\text{move}} = 10$, wealth configuration = LN ($\mu = 0$, $\sigma = 0.5$); Bottom 2nd Left: $\beta_{\text{move}} = 100$, wealth configuration = LN ($\mu = 0$, $\sigma = 0.5$); Bottom 2nd Right: $\beta_{\text{move}} = 1000$, wealth configuration = LN ($\mu = 0$, $\sigma = 0.5$); Bottom Right: $\beta_{\text{move}} = 100{,}000$, wealth configuration = LN ($\mu = 0$, $\sigma = 0.5$).

Chart 2: Segregation at $w_{\text{inc}} = 0$:

If we set $w_{\text{inc}} = 0$ and run the model dynamics, we find that segregation does not obtain at all for even high values of β_{move}. This is attributable to the fact that starting from a reasonably mixed wealth configuration such as the LN ($\mu = 0$, $\sigma = 0.25$), where the initial S is in the region of 72-73% - implying a narrow distribution of cell Potentials (U) across the lattice - that in the absence of a gain function, it does not appear possible for cells to generate any momentum in enhancing their Potential and pulling away from the Potentials of other cells. On the contrary, as the following figure illustrates, even for high β_{move}, we find that S actually increases to around 80%. This suggests that the wealth gain function in conjunction with the cell Potential (U) is

critical in generating the dynamics of RES over time.

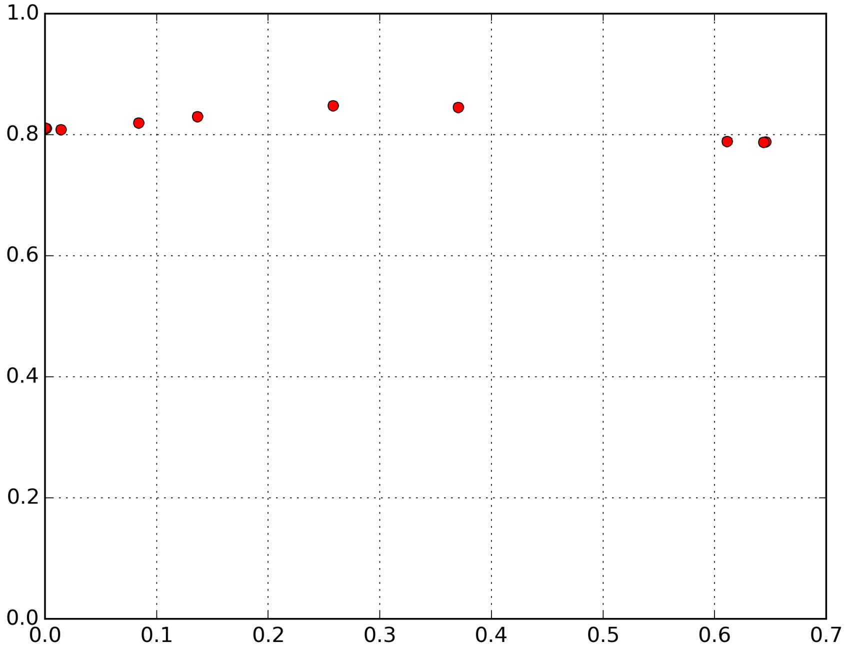

Fig 2. Change in S with ratio of disallowed-realized moves to attempted moves for $w_{\mathbf{inc}} = 0$. Legend: Y-Axis: S = Fraction of population in the richest neighborhoods owning 80% of total city wealth; X-Axis: Ratio of disallowed-realized moves to attempted moves.
